# Contribution of Fat Adjustment to Residual Feed Intake Estimation in Beef Cattle

**DOI:** 10.1111/jbg.70045

**Published:** 2026-02-23

**Authors:** Larissa Bordin Temp, Letícia Pereira, Marcos Jun‐Iti Yokoo, Cintia Righetti Marcondes, Roberto D. Sainz, Fernando Bussiman, Jorge Hidalgo, Daniela Lourenco, Fernando Baldi

**Affiliations:** ^1^ Departamento de Zootecnia Universidade Estadual Paulista (UNESP) Jaboticabal São Paulo Brazil; ^2^ Sustainable Livestock Research Center, Animal Science Institute São José do Rio Preto São Paulo Brazil; ^3^ Embrapa Pecuária Sudeste São Carlos São Paulo Brazil; ^4^ Department of Animal Science University of California Davis California USA; ^5^ Department of Animal and Dairy Science University of Georgia Athens Georgia USA; ^6^ Departamento de Zootecnia Universidade de São Paulo Pirassununga São Paulo Brazil

**Keywords:** *Bos indicus*, feed efficiency, genetic correlation, rump fat thickness

## Abstract

Including fat thickness as a covariate in the regression model used to calculate residual feed intake (RFI) could help preserve carcass quality traits, such as marbling, flavour and juiciness, by accounting for variation in fat deposition. This study aimed to: (1) investigate the benefits of adjusting RFI for rump fat thickness (RFT); (2) estimate variance components and genetic correlations between RFI—calculated with (RFI_F_) and without (RFI_W_) adjustment for RFT—and growth, reproduction and carcass traits using genomic information in beef cattle; and (3) compute accuracy, bias and dispersion of RFI_F_ and RFI_W_ genomic breeding values predicted using single‐step GBLUP (ssGBLUP). We hypothesised that adjusting for RFT would account for a small proportion of RFI variability, and that genetic parameter estimates would support more balanced selection decisions. Phenotypic records were collected from 9094 Nellore animals (3253 females and 5952 males) over 14 feed efficiency tests conducted from 2011 to 2024. The pedigree included 17,407 animals, of which 5812 were genotyped. Linear and threshold animal models were applied for continuous and categorical traits, respectively. Heritability estimates were low for RFI_W_ (0.17) and RFI_F_ (0.16), with a strong genetic correlation between them (0.98), and a weak genetic correlation between RFI_W_ and RFT (0.15). Spearman correlations between RFI_F_ and RFI_W_ breeding values were high: 0.98 in females and 0.95 in males. Genetic correlations of RFI_W_ and RFI_F_ with growth, reproduction and carcass traits ranged from −0.33 to 0.35. Prediction accuracy was similar for RFI_F_ (0.43) and RFI_W_ (0.44), whereas bias (0.00 for RFI_w_ and 0.00 for RFI_F_) and dispersion (0.05 for RFI_w_ and 0.03 for RFI_F_) showed minor differences. Although RFI_F_ captured slightly more genetic variability, the impact was minimal and no differences were observed between RFI_F_ and RFI_W_. The genetic correlations between RFI and traits related to growth, reproduction and carcass were close to zero to moderate, indicating that selection for RFI is unlikely to negatively impact these other traits. However, it is essential to consider the full set of traits in the selection process to avoid potential drawbacks to the overall genetic progress of the herd.

## 1 Introduction

Understanding the biological mechanisms underlying individual variation in feed efficiency is essential for optimising the use of feed resources and mitigating environmental impacts, such as greenhouse gas emissions (Cantalapiedra‐Hijar et al. 2018). Since feed costs can represent up to 75% of the total production expenses, improving feed efficiency is a crucial factor for enhancing the profitability of beef cattle systems (Basarab et al. 2003; Ceacero et al. 2016). Among the developmental stages, the birth‐to‐weaning phase is particularly critical, as it is characterised by superior feed conversion efficiency, which directly enhances the economic viability of breeding and rearing practices in both males and females (Cervieri et al. 2009).

In this context, residual feed intake (RFI), defined as the difference between the observed and predicted feed intake (Koch et al. 1963), stands out as an effective metric to identify animals with below‐average maintenance requirements and feed intake, without compromising performance (Brunes, Baldi, Lopes, Narciso, et al. 2021a; Brunes, Baldi, Lopes, Lobo, et al. 2021b; Sainz et al. 2024). These animals are considered more feed‐efficient, as they require less maintenance energy while sustaining comparable levels of productivity (Sainz et al. 2024), thereby maximising the potential for profitability (Pereira et al. 2023).

One of the main advantages of using RFI as a selection criterion is its phenotypic and genetic independence from production level (Santana et al. 2014). Consequently, selecting animals for low RFI does not lead to increases in mature body weight/size, nor does it elevate maintenance requirements. However, despite these benefits, several studies (Leme and Gomes 2007; Lancaster et al. 2009; Shaffer et al. 2011) have reported potential drawbacks associated with RFI selection. Specifically, animals with negative RFI tend to have leaner carcasses, lower fat deposition rates and reduced carcass weight, which may lead to delayed puberty and reduced reproductive performance. It is important to emphasise that no single trait should be used as the sole selection criterion. Animal identification and selection should be based on a balance between various criteria, as focusing solely on a single trait can result in losses in productivity and economic returns for the other traits in the production system.

To address these concerns, the inclusion of fat thickness as a covariate in the regression model used to calculate the RFI has been proposed as a strategy to mitigate potential unfavourable outcomes of selection (Pereira et al. 2023). This adjustment aims to preserve carcass quality traits, such as marbling, flavour and juiciness, by accounting for variation in fat deposition. While several studies involving *Bos taurus* breeds have evaluated the impact of incorporating fat thickness into RFI models (Schenkel et al. 2004; Basarab et al. 2011; Mao et al. 2013), research on this approach remains scarce in *Bos indicus* breeds, such as Nellore.

Backfat thickness is a highly economically important trait in beef cattle breeding programs, as it is an indicator of carcass quality (Tonussi et al. 2015) and is associated with variations in body composition and energy requirements for maintenance and weight gain. Phenotypically, it is also relevant because it influences the onset of puberty in females. Despite its relevance, little is known about the impact of fat adjustment on RFI estimates in *Bos indicus* cattle, particularly in the Nellore breed. Therefore, our objectives in this study were: (1) to estimate variance components and genetic correlations between RFI with (RFI_F_) and without (RFI_W_) adjustment for rump fat thickness (RFT) and growth, reproduction and carcass traits using genomic information in Nellore cattle; and (2) to compute accuracy, bias and dispersion of RFI_F_ and RFI_W_ genomic estimated breeding values (GEBV) from a single‐step GBLUP evaluation. We hypothesised that fat adjustment would explain only a small portion of the variability in RFI and that the resulting genetic parameter estimates would support more balanced and efficient selection decisions.

## 2 Material and Methods

### 2.1 Data and Traits

Existing data from the Nellore improvement program database of the Brazilian Breeders and Researchers Association (ANCP) were used. Therefore, approval from an institutional ethics committee was not required. The pedigree consisted of 17,407 animals of which 5812 were genotyped using the GGP indicus panel from Neogen. Genotype quality control was performed using preGSF90 software (Misztal et al. 2014; Lourenco et al. 2022), with removal of SNPs or animals with a call rate lower than 0.90, minor allele frequency less than 0.05, a departure from Hardy–Weinberg equilibrium (difference between observed and expected heterozygous frequency) higher than 0.15 or monomorphic SNP. After quality control, 5799 genotyped animals and 42,132 SNP markers were retained for subsequent analyses.

**FIGURE 1:**
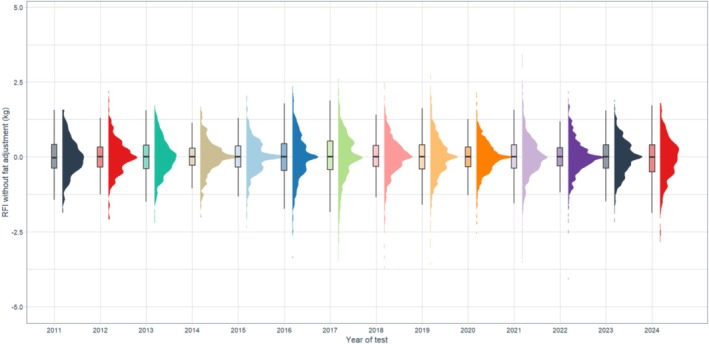
Residual feed intake (RFI) records without adjustment for rump fat thickness across performance test years in Nellore cattle. [Colour figure can be viewed at wileyonlinelibrary.com]

Phenotypic data (collected between 2011 and 2024) for RFI from 9094 Nellore animals (3253 females and 5952 males) were used (14 efficiency test, Figure 1). Two RFI definitions were considered—RFI_W_ (residual feed intake without fat adjustment) and RFI_F_ (residual feed intake adjusted for rump fat thickness), obtained as follows:

(1) $${\mathrm{DMI}}_{i}=\beta_{01}+\beta_{11}{\mathrm{ADG}}_{i}+\beta_{21}{\mathrm{MBW}}_{i}+\varepsilon_{i1},$$

(2) $$\begin{array}{c} {\mathrm{DMI}}_{i}=\beta_{02}+\beta_{12}{\mathrm{ADG}}_{i}+\beta_{22}{\mathrm{MBW}}_{i}+\beta_{32}{\mathrm{RFT}}_{i}+\varepsilon_{i2}, \end{array}$$

where $\varepsilon_{i1}={\mathrm{DMI}}_{i}-{\hat{\mathrm{DMI}}}_{i1}$ represents the RFI_W_, whereas $\varepsilon_{i2}={\mathrm{DMI}}_{i}-{\hat{\mathrm{DMI}}}_{i2}$ represents the RFI_F_; $\beta_{0j}$ represents the intercept; $\beta_{1j}$ represents the regression coefficient of the average daily gain of the *i*th animal (${\mathrm{ADG}}_{i}$); $\beta_{2j}$ represents the regression coefficient of the metabolic body weight of the *i*th animal (${\mathrm{MBW}}_{i}=BW_{i}^{0.75}$); and $\beta_{32}$ represents the regression coefficient of the rump fat thickness of the *i*th animal (${\mathrm{RFT}}_{i}$). It is worth noting that the subscript *j* depicts the model: Equation (1) (*j* = 1) or Equation (2) (*j* = 2). Efficiency tests were conducted according to the guidelines of the manual ‘Procedures for measuring individual feed intake in beef cattle’, consisting of a 21‐day adaptation period followed by a 70‐day testing phase (Mendes et al. 2020). The animals were evaluated under consistent dietary, management and environmental conditions. Heifers began testing immediately after breeding, placing them in the first trimester of gestation. Although the diets followed the same overall formulation each year, their composition varied with seasonal changes in silage and grain quality (Sainz et al. 2024). Feed intake records were excluded from analysis on days when animals were handled offsite for extended periods, during equipment failures or when no feed refusals occurred. Body weights were automatically recorded using full‐body weighing platforms at each water trough (Intergado/Ponta, Belo Horizonte, MG, Brazil).

The average age at the beginning of the performance test was 424.2 ± 126.89 days. Descriptive statistics for back fat thickness (BFT), rib eye area (REA), frame score (FRAME), scrotal circumference adjusted for 365 days of age (SC365), weight adjusted for 365 days (W365) or 450 days of age (W450), age at first calving (AFC), stayability (STAY) and early heifer pregnancy probability (3P) are shown in Table 1.

**TABLE 1:** Descriptive statistics for reproductive, growth, carcass and feed efficiency traits in Nellore cattle.

Traits	Min	Mean ± SD	Max	*N*
RFI_W_ (kg of DMI)	−2.99	−0.0004 ± 1.10	2.76	9094
RFI_F_ (kg of DMI)	−3.05	0.003 ± 1.22	2.91	9094
RFT (mm)	0.28	6.77 ± 3.10	29.91	9094
RFT (mm) ♂	1.18	5.56 ± 1.71	15.18	5952
RFT (mm) ♀	0.28	8.64 ± 3.79	29.92	3253
REA (cm^2^)	30.35	68.99 ± 11.46	126.77	8293
FRAME	−7.20	5.12 ± 2.19	14.38	3202
SC365 (cm)	17.50	24.42 ± 2.70	33.90	2879
W365 (kg)	183	331.35 ± 52.15	537	5097
W450 (kg)	203	384.48 ± 59.27	617	5113
AFC (months)	20	24.27 ± 3.81	49	2211
STAY	1	1.54 ± 0.49	2	1516
3P	1	1.92 ± 0.26	2	1841

*Note:* ♀: females; ♂: males.

Abbreviations: 3P, early heifer pregnancy probability [heifers who became pregnant by 20 months of age were classified as precocious (success: 2), otherwise, as conventional (failure: 1)]; AFC, age at first calving; FRAME, frame score; *N*, Number of records; REA, rib eye area; RFI_F_, residual feed intake adjusted for rump fat thickness; RFI_W_, residual feed intake without adjustment for rump fat thickness; RFT, rump fat thickness; SC365, adjusted scrotal circumference (cm) at 365 days; SD, standard deviation; STAY, stayability [dams with at least three calvings by 76 months of age, were classified as success (2), otherwise as failure (1)]; W365, adjusted weight at 365 days; W450, adjusted weight at 450 days.

Carcass traits (BFT, RFT and REA) were measured at the end of the efficiency tests using ALOKA 500V equipment with a 3.5 MHz linear probe, according to BIF (2018) guidelines. The animals were scanned for Longissimus muscle area (LMA) and backfat (BFT) between the 12th and 13th ribs at the LM, and rump fat (RFT) was measured at the junction of the Gluteus medius and Biceps femoris muscles, between the ileum and ischium bones. All equipment, software and technicians were certified by either the Ultrasound Guidelines Council (UGC; ultrasoundguidelinescouncil.org/) or the Brazilian Ultrasound Technicians Association (ATUBRA).

We used the FRAME classification proposed by Guimaraes (2020) for Nellore cattle, as it was developed using data from herds participating in the ANCP. Briefly, it was based on multiple linear regressions considering the relationship between height (HH), REA, BFT, RFT, sex (male—M, or female—F) and age (AGE) as follows:

(3) $$\begin{array}{c} {\text{FRAME}}_{M_{i}}=-20.35+0.1305{\mathrm{REA}}_{i}+0.2633{\mathrm{BFT}}_{i}-0.5901{\mathrm{RFT}}_{i}\\ +0.1139{\mathrm{HH}}_{i}+0.0056{\mathrm{AGE}}_{i}, \end{array}$$

(4) $$\begin{array}{c} {\text{FRAME}}_{F_{i}}=-11.87+0.1316{\mathrm{REA}}_{i}-0.2457{\mathrm{BFT}}_{i}-0.6218{\mathrm{RFT}}_{i}\\ +0.1139{\mathrm{HH}}_{i}+0.0009507{\mathrm{AGE}}_{i}, \end{array}$$

where ${\text{FRAME}}_{M_{i}}$ and ${\text{FRAME}}_{F_{i}}$ are the FRAME for the *i*th male or the *i*th female, respectively.

Scrotal circumference (SC365) was measured in centimetres with a measuring tape, from 9 to 18 months of age in intervals of 3 months, followed by a linear adjustment for 365 days. Weight was recorded every 90 days until 18 months of age, and then annually up to 16 years. Age adjustment followed Garnero et al. (2001), using the average daily gain (kg/day) of each period to standardise weights.

Age at first calving (AFC) was simply the age in months at the cows' first calving. Stayability (STAY) was defined as the ability of dams to produce at least three calves by 76 months of age. Those meeting this criterion were classified as success (2); otherwise, as failure (1). The probability of precocious calving (3P) was evaluated by exposing heifers to mating at 10–14 months of age during a 3‐month breeding season. Those that did not conceive were re‐exposed at 24 months. Heifers that calved by 30 months were classified as success (2); otherwise as failure (1).

### 2.2 Variance Components Estimation

A linear animal model was used to estimate (co)variance components and genetic parameters for RFI_W_, RFI_F_, REA, FRAME, SC365, W365, W450 and AFC as follows:

(5) $$\begin{array}{c} y=\mathrm{X\beta}+\mathrm{Zu}+e, \end{array}$$

where y is the vector of phenotypic observations; β is the vector of systematic effects (contemporary groups—CG—defined by farm, management group, sex, year and season of birth); and age at the start of the feed efficiency test (covariate for RFI_W_ and RFI_F_) treated as fixed in the linear model; u is the vector of random animal additive effects; e is the vector of random residuals; and X and Z are incidence matrices relating the effects in β and u to y.

For 3P and STAY a threshold model was implemented via Bayesian inference, considering the systematic effects of CG and the random animal additive effects. Following Equation (5), replacing y by the liability for those traits the model becomes:

(6) $$\begin{array}{c} l=\mathrm{X\beta}+\mathrm{Zu}+e, \end{array}$$

where l is the vector of unobserved liabilities; β, u, e, X and Z were defined in Equation (5). Given the model parameters, the elements of l are assumed conditionally independent, distributed as $\left.l\right|\beta ,u,A,\sigma_{\beta }^{2},\sigma_{u}^{2},\sigma_{e}^{2}\sim \mathrm{MVN}\left\{\mathrm{X\beta}+\mathrm{Zu},I\sigma_{e}^{2}\right\}$, with $\sigma_{e}^{2}=1$ to make the model identifiable (Sorensen and Gianola 2002). It was also assumed that: $\left.\beta \right|I,\sigma_{\beta }^{2}\sim \mathrm{MVN}\left\{0,I\sigma_{\beta }^{2}\right\}$; $\left.u\right|A,\sigma_{u}^{2}\sim \mathrm{MVN}\left\{0,H\sigma_{u}^{2}\right\}$ and $\left.e\right|I,\sigma_{e}^{2}\sim \mathrm{MVN}\left\{0,I\sigma_{e}^{2}\right\}$, with H being the joint covariance matrix for non‐genotyped and genotyped animals (Legarra et al. 2009), and I an identity matrix of proper order. Prior distributions for the variance components were $\sigma_{\beta }^{2}=1.00$, $\left.\sigma_{u}^{2}\right|\nu_{u}\sim \chi^{-2}\left(\nu_{u},S_{u}^{2}\right)$ and $\left.\sigma_{e}^{2}\right|\nu_{e}\sim \chi^{-2}\left(\nu_{e},S_{e}^{2}\right)$, being $\chi^{-2}$ an inverted chi‐square distribution with ν degrees of belief and scale parameter S.

For 3P and STAY, $\sigma_{u}^{2}$ and $\sigma_{e}^{2}$ were estimated via Gibbs sampling as implemented in the GIBBSf90+ software (Misztal et al. 2014) using the default prior for random and systematic effects (i.e., a Gaussian prior for random effects, and a flat prior for systematic effects). A single chain containing 1,000,000 samples was generated. The first 50,000 samples were discarded as burn‐in, and one in every 100 samples were stored; thus, inference was made over 9500 samples from the posterior distribution. Convergence was checked by visual inspection of the generated chain and by the Geweke's test (Geweke 1992), the Heidelberg and Welch test (Heidelberger and Welch 1981) and the Raftery and Lewis test (Raftery and Lewis 1992), implemented in the ‘*boa*’ R package (Smith 2007). The heritability estimates were classified as low (< 0.20), moderate (from 0.20 to 0.40) and high (> 0.40) as proposed by Bourdon (1997).

Genetic correlations were estimated using three‐trait models. For all analyses, RFI_W_ and RFI_F_ were consistently included, whereas the third trait (REA, FRAME, SC365, W365, W450, AFC, STAY or 3P) varied among the analyses. For linear traits, genetic correlations were estimated using restricted maximum likelihood (EM‐REML) via the BLUPf90+ software. For threshold traits, Bayesian inference was performed using the GIBBSf90+ software (Lourenco et al. 2022). Genetic correlation estimates were classified as low (< 0.30), moderate (from 0.30 to 0.70) and high (> 0.70) according to Hill (2013).

### 2.3 GEBV and Validation

Single‐trait models were used to predict the GEBV for RFI_W_ and RFI_F_. Under multivariate normality, the (co)variance matrix for the random effects was given by

(7) $$\begin{array}{c} \mathrm{Var}\left[\begin{array}{c} u\\ e \end{array}\right]=\left[\begin{array}{cc} H\sigma_{u}^{2} & 0\\ 0 & I\sigma_{e}^{2} \end{array}\right], \end{array}$$

where H is the joint covariance matrix for non‐genotyped and genotyped animals (Legarra et al. 2009), whose inverse was computed as in Aguilar et al. (2010); I is an identity matrix of proper order; $\sigma_{u}^{2}$ is the additive genetic variance; and $\sigma_{e}^{2}$ is the residual variance. The genomic relationship matrix (G) was constructed as in VanRaden (2008) (method 1). To avoid singularity issues, G was blended with 5% of the pedigree‐based relationship matrix for genotyped animals ($A_{22}$). Incompatibility issues were addressed by tuning G as in Vitezica et al. (2011).

The validation method used was the linear regression (Legarra and Reverter 2018). The whole dataset (subscript *w*) included all sources of information (phenotypes, genotypes and pedigrees) for all animals (*N* = 5443). The partial dataset (subscript *p*) does not include phenotypes for the last 2 years of data (*N* = 4297). This was done to ensure enough focal animals for validation. Focal animals (*N* = 1146) were young, genotyped individuals without their own (or progeny) records in the partial dataset. The whole data set can be interpreted as the posterior confirmation of the validity of the selection decisions. In contrast, the partial data set represents the evaluation conducted during selection decisions (Macedo et al. 2020). The validation statistics were prediction accuracy (acc), bias (δ), dispersion (*b*_*1*_) and correlation (corr), computed as follows:

(8) $$\begin{array}{c} \mathrm{acc}=\sqrt{\frac{\mathrm{cov}\left({\hat{u}}_{w},{\hat{u}}_{p}\right)}{\left(1-\bar{F}\right){\hat{\sigma }}_{u}^{2}}}, \end{array}$$

(9) $$\begin{array}{c} \delta =\frac{{\bar{\hat{u}}}_{p}-{\bar{\hat{u}}}_{w}}{{\hat{\sigma }}_{u}}, \end{array}$$

(10) $$\begin{array}{c} b_{1}=\frac{\mathrm{cov}\left({\hat{u}}_{w},{\hat{u}}_{p}\right)}{\mathrm{var}\left({\hat{u}}_{p}\right)}, \end{array}$$

where ${\hat{u}}_{w}$ is the vector of GEBV from the whole dataset; ${\hat{u}}_{p}$ is the vector of GEBV from the partial dataset; $\bar{F}$ is the average pedigree‐inbreeding coefficient for focal animals; ${\hat{\sigma }}_{u}^{2}$ is the estimated additive genetic variance; and ${\bar{\hat{u}}}_{p}$ is the average of ${\hat{u}}_{p}$ (likewise for ${\bar{\hat{u}}}_{w}$).

## 3 Results and Discussion

The *R* ^2^ for the model without fat was 0.2337, while the *R* ^2^ for the model adjusted for fat was 0.2287. These values indicate that both models explain a similar proportion of the phenotypic variance in feed intake, with only a marginal change in explained variance when the fat term is included. Heritability estimates (Table 2) ranged from 0.13 to 0.42. A low estimate was observed for AFC (0.13), whereas STAY (0.20) and 3P (0.29) presented moderate values. However, it is important to note that these estimates had high uncertainty, likely due to our relatively small sample size. The limited dataset may have biased the variance components, while the similar environmental conditions under which the animals were evaluated might have reduced the residual variance, proportionally increasing the heritability. These factors likely explain the moderate heritability estimates observed, despite the generally low values found in literature. For example, Negreiros et al. (2024) reported low heritability estimates for 3P (0.16), STAY (0.13) and AFC (0.08) in Nellore cattle. Similarly, other studies have consistently shown low heritability for these traits (Bonamy et al. 2019; Silva Neto et al. 2020; Kluska et al. 2018; Schmidt et al. 2018).

**TABLE 2:** Estimates of variance components and heritabilities for traits related to feed efficiency, carcass, growth and reproduction in Nellore cattle.

Traits	$\sigma_{a}^{2}$ ± SD	$\sigma_{e}^{2}$ ± SD	*h* ^2^ ± SD
RFI_W_	0.08 ± 0.01	0.39 ± 0.01	0.17 ± 0.02
RFI_F_	0.07 ± 0.01	0.38 ± 0.01	0.16 ± 0.02
REA	13.31 **±** 1.12	33.23 **±** 0.82	0.28 **±** 0.02
FRAME	0.83 **±** 0.10	1.66 **±** 0.07	0.33 **±** 0.03
SC365	1.77 **±** 0.15	2.49 **±** 0.13	0.42 **±** 0.03
W365	251.20 **±** 30.54	637.36 **±** 23.61	0.28 **±** 0.03
W450	319.13 **±** 34.45	644.86 **±** 25.16	0.33 **±** 0.03
AFC	1.47 **±** 0.35	9.84 **±** 0.41	0.13 **±** 0.03
STAY*	0.27 (0.04; 0.52)	1.02 (0.91; 1.12)	0.20 (0.06; 0.35)
3P*	0.45 (0.03; 0.87)	1.00 (0.91; 1.09)	0.29 (0.11; 0.48)

Abbreviations: *HPDI, high posterior density interval; 3P, early heifer pregnancy probability [heifers who became pregnant by 20 months of age were classified as precocious (success: 2), otherwise, as conventional (failure: 1)]; AFC: age at first calving; FRAME, frame score; REA, rib eye area; RFI_F_, residual feed intake adjusted for rump fat thickness; RFI_W_: residual feed intake without adjustment for rump fat thickness; RFT: rump fat thickness; SC365, adjusted scrotal circumference (cm) at 365 days; SD, standard deviation; STAY, stayability [dams with at least 3 calvings by 76 months of age, were classified as success (2), otherwise as failure (1)]; W365, adjusted weight at 365 days; W450, adjusted weight at 450 days.

Moderate heritability estimates were obtained for REA, FRAME, APM, W365 and W450, ranging from 0.28 (REA) to 0.33 (FRAME; W450) (Table 2). Similar results have been reported in previous studies. Negreiros et al. (2024) found moderate estimates for FRAME (0.35), W450 (0.39) and REA (0.37), while Negreiros et al. (2022) observed moderate values for FRAME (0.30) and W450 (0.26). Likewise, working with Charolais and Charbray cattle breeds, Ríos‐Utrera et al. (2018) reported a moderate heritability estimate for FRAME score (0.25). Consistency across studies may reflect the genetic background of Nellore cattle, which generally exhibits moderate additive variance for growth and size‐related traits, as well as the use of similar age‐adjusted weight records. Overall, these results reinforce that selection based on these traits is feasible.

A high heritability estimate was observed for SC365 (0.42), indicating that a higher proportion of the variation among animals is due to additive genetics in this trait compared to the other studied traits. This estimate is within the previously reported range for the Nellore breed (0.33–0.48), as documented by Carvalho Filho et al. (2020), Kluska et al. (2018) and Negreiros et al. (2022).

Heritability estimates for RFI_W_ and RFI_F_ were low (0.16 and 0.17), highlighting the substantial influence of environmental effects on these traits. Using random regression models, Ramos et al. (2024) reported posterior heritability estimates for RFI ranging from 0.03 to 0.26 in Nellore cattle during days 1 to 84 of the performance test, reinforcing the importance of environmental effects on feed efficiency, particularly with respect to management and feedlot conditions. Additionally, low to moderate estimates of genetic variance (0.02–0.27) for RFI were also found by Grion et al. (2014; Nellore), Cancino‐Baier et al. (2019; Guzerat), Oliveira et al. (2022; Nellore bulls), Brunes et al. (2023; Nellore), Pereira et al. (2023; Guzerat) and Sainz et al. (2024; Nellore) in several Brazilian cattle populations. This suggests that RFI has a low to moderate heritability in Nellore cattle, contrasting with taurine breeds (e.g., Angus [0.30–0.40; Torres‐Vázquez et al. 2018; Duff et al. 2021] and various continental breeds [0.50; Esfandyari and Jensen 2021]), indicating that faster growing breeds have a higher heritability for feed efficiency than well‐adapted to local tropical conditions. Another relevant aspect to consider is that heritability estimates tend to vary depending on the statistical model used to predict RFI, as well as on the population structure analysed and the methodology applied to measure the trait.

Including body fat measures in the model to determine RFI aims to ensure that differences in RFI are not merely due to fatness, while also reducing the risk of selecting for excessively lean carcasses in slaughter cattle or compromising body condition in replacement heifers (Basarab et al. 2003; Kelly et al. 2019). Crews (2005) emphasised that the composition of weight gain is influenced by the age at which animals are assessed, noting that fat deposition requires more energy than protein deposition, whereas maintaining fat tissue is less energetically demanding than maintaining protein tissue. By including fat thickness in the DMI prediction model, Ceacero et al. (2016) observed a reduction in the heritability estimates for RFI, from 0.24 (RFI_W_) to 0.20 (RFI_F_), and to 0.22 when both BFT and RFT were considered. This suggests that some of the genetic variation in feed efficiency may arise from differences in weight gain, especially from the composition of that gain.

The genetic correlation between RFI_W_ and RFI_F_ was strong (Table 3), with values above 0.96, indicating that including RFT as a covariate in the model has little impact on RFI in this population and the genetic factors influencing RFI at earlier developmental stages and lower body weights remain consistently active throughout the animal's growth. RFI adjusted for production and for both production and BFT showed a high within‐breed genetic correlation (0.99). However, adjusting for fat changed the breed rankings for RFI. Angus and Hereford improved significantly after fat adjustment, while Blonde d'Aquitaine and Limousin remained the most efficient breeds regardless of fat adjustment (Schenkel et al. 2004). Jiu et al. (2019) reported similar results using Pearson's correlation coefficients. These authors observed a strong, positive correlation (*r* = 0.99, *p* < 0.0001) between adjusted RFI (BFT and final ultrasound backfat) and unadjusted RFI in Angus, Charolais and Angus‐crossbred steers (Angus bulls with a hybrid dam line—Kinsella composite cattle).

**TABLE 3:** Genetic and phenotypic correlation between residual feed intake definitions and rump fat thickness in Nellore cattle.

Models	$r_{g}$ ± SD	$r_{p}$ ± SD
RFI_W_ − RFI_F_	0.98 ± 0.03	0.98 ± 0.00
RFI_W_ − RFI_F_*	0.99 ± 0.00	0.98 ± 0.00
RFI_W_ − RFI_F_**	0.96 ± 0.00	0.97 ± 0.00
RFI_W_ − RFT	0.15 ± 0.08	0.03 ± 0.01

*Note:* *: females; **: males.

Abbreviations: $r_{g}$, genetic correlation; $r_{p}$, phenotypic correlation; SD, standard deviation.

The genetic correlation between RFI_W_ and RFT was weak (0.15), and the phenotypic correlation was 0.03. These findings suggest that improvements in feed efficiency during the post‐weaning phase can be achieved independently of changes in fat deposition. This independence allows for greater flexibility in selection strategies without compromising carcass fatness traits.

In Nellore cattle, Ceacero et al. (2016) reported moderate genetic correlations between RFI and RFT (0.30) and BFT (0.37). Similarly, Berry and Crowley (2013) found a low correlation of 0.20 between RFI and carcass fat thickness. Arthur et al. (1996, 2001) reported genetic correlations of 0.17 for rib fat depth and 0.24 for overall fat depth in British and Angus breed bulls. Those results suggest that selection for enhanced feed efficiency is often associated with reduced fat deposition. This relationship is particularly relevant because fat cover contributes not only to carcass composition and reproduction but also to meat quality traits. In this regard, Malheiros et al. (2015) emphasised that leaner carcasses may pose challenges for tenderness, since Nellore cattle with higher BFT demonstrated superior meat tenderness compared to those with lower fat accumulation.

Overall, while these literature findings indicate that the genetic correlation between RFI_W_ and RFT is not strong and that an antagonistic correlated response may not pose a major concern, they underscore the importance of exercising caution and carefully designing the breeding program targeting simultaneous selection for these economically important traits using a properly defined selection index.

The observed differences in BFT and RFT may be associated with breed origin, particularly between *Bos taurus* and *Bos indicus* , the latter being better adapted to tropical environments (Barwick et al. 2009). Johnston et al. (2003) observed that tropical genotypes tend to exhibit greater subcutaneous rump fat (P8) relative to rib fat than temperate genotypes. Supporting these findings, Barwick et al. (2009) demonstrated that Brahman cattle showed higher fat deposition over the rump and similar rib fat thickness compared to Tropical Composite cattle after an extended period grazing pastures of variable quality.

**FIGURE 2:**
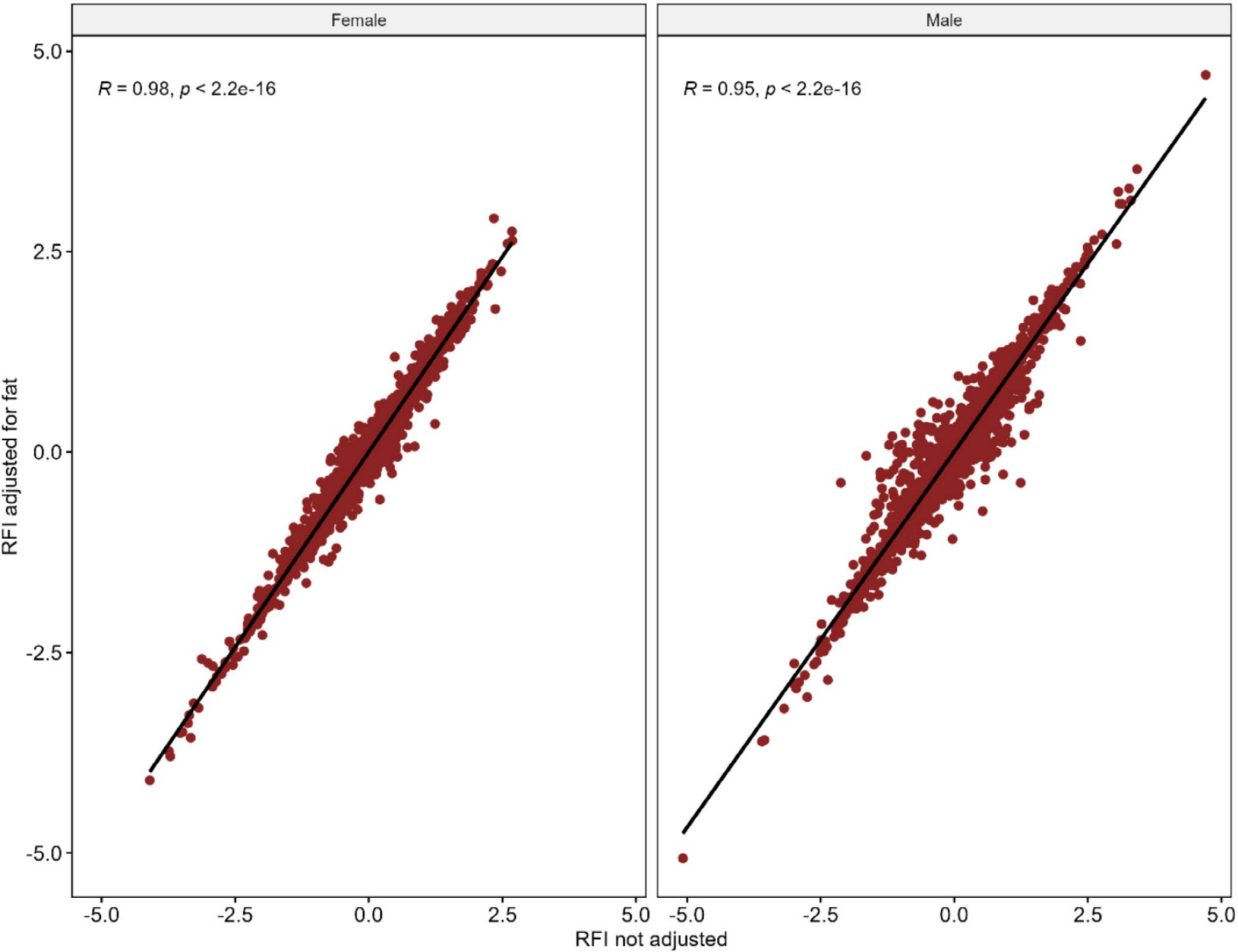
Spearman's correlations between genomic breeding values for residual feed intake, with and without adjustment for rump fat thickness, in male and female Nellore cattle. [Colour figure can be viewed at wileyonlinelibrary.com]

**FIGURE 3:**
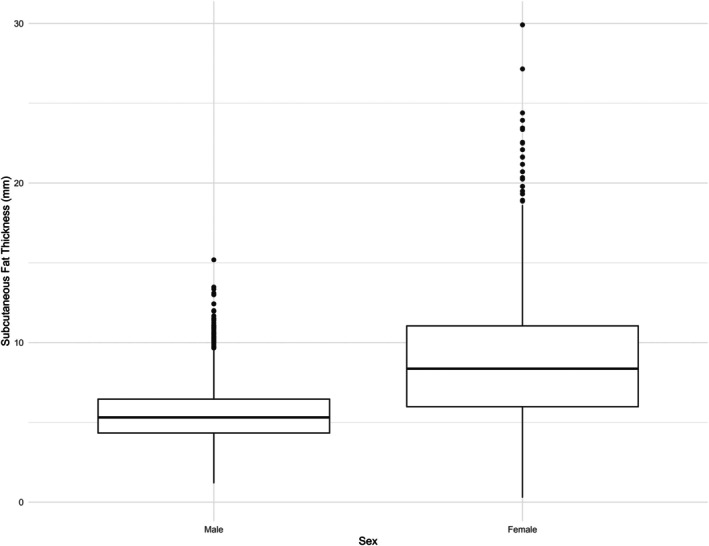
Comparison of rump fat thickness deposition between male and female Nellore cattle.

In line with the results presented in Table 3, Figure 2 provides complementary information, showing correlation between RFI_F_ and RFI_W_ breeding values by sex. High phenotypic correlations were observed for both females (*r* = 0.98) and males (*r* = 0.95), with highly significant *p* values (*p* < 2.2 × 10^−16^). The strong linear relationship between adjusted and unadjusted RFI values suggests that RFT explains little additional variation in the model, especially in females. Therefore, adjusting RFI for RFT may not be essential for selection purposes, depending on the breeding goals and population structure. In cow–calf production systems, fat reserves play a crucial role for females, as they are essential for simultaneously supporting body maintenance, pregnancy and future lactation demands (Santana et al. 2014). This physiological requirement helps explain the results observed in Figure 3, where females showed substantially higher fat deposition compared to males.

Herd and Arthur (2009) reported that body composition accounts for up to 5% of the genetic variability in RFI, while Hill and Ahola (2012) suggested including carcass traits such as REA and BFT in RFI prediction models. This inclusion aims to adjust feed intake according to body composition and allow comparisons between animals at different stages of the growth curve. Both studies were conducted with *Bos taurus* (European) cattle, in which BFT (typically measured in the rib region) is commonly used. This fat depot tends to accumulate later in the finishing phase. In contrast, RFT, selected in the present study for RFI adjustment, is deposited earlier in the growth period.

From the slaughterhouse perspective, carcass yield is more strongly influenced by REA and fat deposition, particularly in strategic locations such as the rump and rib regions. Schenkel et al. (2004) reported that BFT were different between growing Angus, Hereford, Limousin, Charolais and Simmental bulls. It is common to see breed included in the determination of RFI as a fixed effect when the dataset includes several breeds (Lawrence et al. 2011).

Genetic correlations between RFI_W_ or RFI_F_ and growth, reproductive and carcass traits ranged from −0.33 to 0.35 (Table 4). The estimated genetic correlations between RFI and REA were close to zero in both models, indicating no relevant genetic association between these traits and that selection to improve RFI would not affect REA (Table 4). For FRAME, an increase in the estimated genetic correlation was observed, rising from 0.04 with RFI_W_ to 0.14 with RFI_F_. This indicates that when RFI is adjusted for fat thickness, larger‐framed animals tend to be less efficient. Regarding growth traits, W365 (0.15; 0.18) and W450 (0.23; 0.27) showed positive and weak genetic correlations with RFI_w_ and RFI_F_, respectively.

**TABLE 4:** Genetic correlations between residual feed intake, with (RFI_F_) and without adjustment (RFI_W_) for rump fat thickness, and traits related to feed efficiency, carcass, growth and reproduction in Nellore cattle.

Paired trait	RFI_W_ ± SD	RFI_F_ ± SD
REA	−0.05 **±** 0.07	−0.02 **±** 0.07
FRAME	0.04 **±** 0.04	0.14 **±** 0.04
SC365	0.34 **±** 0.10	0.35 **±** 0.11
W365	0.15 **±** 0.11	0.18 **±** 0.12
W450	0.23 **±** 0.10	0.27 **±** 0.11
AFC	0.25 **±** 0.15	0.28 **±** 0.18
STAY*	0.21 (−0.24; 0.67)	0.12 (−0.33; 0.58)
3P*	−0.33 (−0.83; 0.15)	−0.24 (−0.75; 0.27)

Abbreviations: *HDPI, high posterior density interval; 3P, early heifer pregnancy probability [heifers who became pregnant by 20 months of age were classified as precocious (success: 2), otherwise, as conventional (failure: 1)]; AFC, age at first calving; FRAME, frame score; REA, rib eye area; RFI, residual feed intake; RFI_F_, residual feed intake adjusted for rump fat thickness; RFI_W_, residual feed intake without adjustment for rump fat thickness; SC365, adjusted scrotal circumference (cm) at 365 days; SD, standard deviation; STAY, stayability [dams with at least 3 calvings by 76 months of age, were classified as success (2), otherwise as failure (1)]; W365, adjusted weight at 365 days; W450, adjusted weight at 450 days.

In practical terms, the weak genetic association between RFI and REA, FRAME and growth indicates that selection for RFI, as a complementary criterion, contributes to reducing feed consumption without compromising carcass, growth and body composition traits. Similarly, Grion et al. (2014), Santana et al. (2014), Ceacero et al. (2016), Moraes et al. (2016), Brunes, Baldi, Lopes, Narciso, et al. (2021a), Brunes, Baldi, Lopes, Lobo, et al. (2021b) and Brunes et al. (2023) reported low genetic correlation estimates between RFI_W_ and reproductive, growth and carcass traits in Nellore cattle. Moraes et al. (2016) reported a negative genetic correlation (−0.70) between RFI adjusted for overall fat cover (0.35 BFT + 0.65 RFT) with REA, indicating that selecting more feed‐efficient animals could result in animals with larger REA. The authors also noted that the negative correlation observed between fat‐adjusted RFI and carcass traits may be attributed to the limited number of animals evaluated, a similar situation to that of the present study, which includes fewer than 10,000 animals.

Correlations between RFI and STAY were positive (Table 4); however, their confidence intervals encompassed zero, indicating that these correlations were not significantly different from zero. Comparable findings were reported by Brunes et al. (2023) and Sainz et al. (2024), who observed genetic correlations of 0.17 and 0.15 for RFI_W_ in Nellore cattle. These low estimated genetic correlations indicate that selection for RFI would not strongly compromise reproductive efficiency; however, priority should be given to animals with a balance between reproductive and feed efficiency traits through a selection index accounting for the correlation among traits.

In a breeding herd, the use of RFI as a complementary selection criterion can be beneficial for females reared in extensive systems with limited feed supply (Brunes, Baldi, Lopes, Narciso, et al. 2021a; Brunes, Baldi, Lopes, Lobo, et al. 2021b). The adoption of RFI is strategic due to the high and favourable genetic correlation with energy requirements (Sainz et al. 2024). Thus, more efficient females tend to have lower energy demands for maintenance, ensuring productive and reproductive efficiency even under restricted nutritional conditions.

The correlations with 3P tended to be negative in both models; however, as their credible intervals included zero, they were not significantly different from zero, indicating no clear evidence of genetic antagonism between feed efficiency and the reproductive ability of females to achieve three calvings by 30 months of age. Sainz et al. (2024) reported a genetic correlation of −0.11, whereas Brunes, Baldi, Lopes, Narciso, et al. (2021a) and Brunes, Baldi, Lopes, Lobo, et al. (2021b) observed a value of 0.28 in Nellore cattle. However, the wide confidence intervals (including zero) for both reproductive traits highlight the need for cautious interpretation and reinforce the importance of further studies with larger datasets. In this context, breeding programs that simultaneously adopt sexual precocity and RFI as criteria should prioritise balanced animals for both traits, ensuring gains in feed efficiency without compromising sexual precocity and fertility.

The findings of our study are consistent with those of Santana et al. (2014) and Abreu et al. (2019), who reported near‐zero genetic correlations between REA and RFI, suggesting that selection for feed efficiency is unlikely to negatively impact carcass traits. Baker et al. (2006), Zorzi et al. (2013) and Ahola et al. (2011) working with Angus steers, Nellore and Angus bulls, reported that selection for low RFI did not influence BFT and REA. This finding supports the concept that feed efficiency does not compromise carcass value, as BFT and REA appear to be more strongly influenced by nutritional management than by energy requirements themselves.

The prediction accuracy was similar for RFI_W_ (0.44) and RFI_F_ (0.43). Bias (−0.00 for RFI_w_ and −0.00 for RFI_F_) and dispersion (0.05 for RFI_w_ and 0.03 for RFI_F_) had minor differences. Following the LR statistics, RFI_F_ showed slightly greater potential for genetic gain, although the difference was also small. Brunes et al. (2023), working with RFI in both a complete population and test‐separated groups of Nellore cattle, also reported no differences in prediction ability between models, consistent with findings in our study and studies by Silva et al. (2016) and Brunes et al. (2022). Based on the results of the present study, it can be reinforced that using RFI_F_ rather than RFI_W_ does not lead to relevant differences in the estimation of variance components, genetic correlations or predictive ability.

## 4 Conclusion

Our findings suggest that adjusting for RFT would account for a small portion of the variability in RFI, as no differences in terms of genetic parameters and prediction accuracy are observed between adjusted and unadjusted RFI. The correlations between RFI and traits related to growth, reproduction and carcass are close to zero to moderate, indicating that selection for RFI is unlikely to negatively impact these other traits. However, it is essential to consider the full set of traits in the selection process to avoid potential drawbacks to the overall genetic progress of the herd.

## Funding

This work was supported by Fundação de Amparo à Pesquisa do Estado de São Paulo (2024/17795‐0).

## Conflicts of Interest

The authors declare no conflicts of interest.

## Data Availability

The data that support the findings of this study are available on request from the corresponding author. The data are not publicly available due to privacy or ethical restrictions.
